# Genetic diversity and population structure of early-maturing tropical maize inbred lines using SNP markers

**DOI:** 10.1371/journal.pone.0214810

**Published:** 2019-04-09

**Authors:** Gloria Boakyewaa Adu, Baffour Badu-Apraku, Richard Akromah, Ana Luisa Garcia-Oliveira, Frederick Justice Awuku, Melaku Gedil

**Affiliations:** 1 CSIR-Savanna Agricultural Research Institute, Tamale, Ghana; 2 International Institute of Tropical Agriculture (UK) Limited, Carolyn House, Croydon, United Kignodm; 3 Department of Crop and Soil Sciences, Faculty of Agriculture, Kwame Nkrumah University of Science and Technology, Kumasi, Ghana; 4 International Institute of Tropical Agriculture (IITA), Ibadan, Nigeria; University of Helsinki, FINLAND

## Abstract

Information on genetic diversity and population structure are very important in any breeding programme for the improvement of traits of interest and the development of outstanding products for commercialization. In the present study, we assessed the genetic diversity of 94 early-maturing white and yellow tropical maize inbred lines using single nucleotide polymorphism (SNP) markers. The larger number of SNP markers used in this study allowed a clearer inference of the population structure of the 94 inbred lines. Cluster analysis resolved the inbred lines into different clusters based on their pedigree, selection history and endosperm colour. However, three heterotic groups were revealed by population structure analysis, but additional field evaluation could be more informative to confirm the heterotic groups identified. Nevertheless, wide genetic variability existed among the inbred lines making them unique with the potential to contribute new beneficial alleles to maize breeding programmes in the tropics, especially in the West and Central Africa (WCA) sub-region.

## Introduction

For good progress from selection in any crop improvement programme, information on the genetic diversity and population structure of the base germplasm is crucial. Therefore, plant breeders routinely resort to newly available tools to make informed decisions on selection. In the past, a number of researchers have highlighted the importance and the need for accurate assessment of genetic diversity in applied breeding programmes. This allows the examination of issues relating to the assignment of inbred lines to heterotic groups, selection of efficient testers for testing of inbred lines in hybrid combinations, drawing inferences on the introgression of desirable genes from diverse germplasm sources into available genetic base, identification of diverse parental combinations to create segregating progenies with maximum genetic variability for further selection and the estimation of genetic diversity loss during conservation or selection [1–6]. Studies of the early and extra-early maize germplasm in sub-Saharan Africa (SSA) have shown conclusively that genetic diversity is of primary importance in the display of heterosis. Inbred lines from different heterotic groups produced higher-yielding hybrids than those of lines from within the same heterotic group [7]. Therefore, the assessment of genetic diversity within and between plant populations is routinely carried out via different marker techniques such as morphological, biochemical and molecular markers [8,9].

DNA based markers are preferred over morphological and biochemical markers because they are not affected by environmental factors and/or by the developmental stage of the plant [8,10]. Consequently, DNA markers have been an indispensable tool for characterizing genetic resources and providing breeders with more detailed information to assist in selecting diverse parents [11]. In the past, various molecular markers including simple sequence repeats (SSRs) have been extensively used in maize to study the correlation between genetic distance and hybrid performance, heterosis, combining ability and assign inbred lines into heterotic groups [12–17]. For example, forty-two early maturing inbred lines were genotyped by Oyekunle, Badu-Apraku, Hearne, and Franco [17] using 23 SSR markers and grouped them into five heterotic groups. The information generated also provided a better understanding of the genetic relationships among the early-maturing inbred lines and facilitated more effective utilization of the inbred lines in the breeding programme for the development of synthetic varieties and hybrids, and formation of heterotic populations used to derive promising inbred lines. However, the choice of either one or the other marker type, the number of loci, the cost of marker as well as the purpose of the study and the evolutionary history of the populations under investigation play a major role [18].

With the advances in marker technology, single nucleotide polymorphism (SNP) markers have become the choice due to their low cost per data point, high genomic abundance, locus-specificity, co-dominance, the potential for high throughput analysis and lower genotyping error rates [6,19–24]. Studies on the comparative utilization of either SSRs or SNPs have revealed that SSRs with moderate density seems to be more effective for diversity and population structure analysis in maize but as the number of SNP markers increases the results obtained are comparable [19,25].

In the 1990s, the maize improvement programme of IITA initiated a breeding programme for the extra-early- and early-maturing groups which resulted in the development of some multiple stress tolerant (drought, *Striga*, and streak resistant/tolerant) broad-based populations such as TZE-W Pop DT STR and TZE-Y Pop DT STR in the early maturing (90–95 days to physiological maturity) group, and TZEE-W Pop DT STR and TZEE-Y Pop DT STR in the extra-early (80–85 days to physiological maturity) maturing group during the first decade of the inception of the programme [26,27]. Subsequently, numerous open-pollinated varieties (OPVs) and inbred lines have been extracted from these multiple stress tolerant broad-based populations and their derivatives for all agro-ecologies of West and Central Africa (WCA) sub-regions by IITA and its National Agricultural Research (NARS) partners, which has ultimately played an important role in hybrid maize development in WCA. Most of these inbred lines have been characterized using both morphological and/or molecular markers especially SSR markers [28]. Given the cost-effectiveness of SNP markers and their growing use for a wide range of applications in different crops, it is of interest to shift to next-generation markers in the assessment of genetic differences and relationships among the new extra-early- and early-maturing white and yellow maize inbred lines that have been recently developed by IITA for their effective classification into heterotic groups. This will serve as a guide to parent selection for further hybrid development. Therefore, the objective of the present study was to examine the genetic diversity and population structure of *Striga* resistant and/or drought-tolerant maize inbred lines derived from different source populations selected from the panel of inbred lines of the IITA maize improvement programme using SNP markers.

## Materials and methods

### Germplasm

The 94 inbred lines used in this study were developed by the West and Central Africa Collaborative Maize Research Network/International Institute of Tropical Agriculture (WECAMAN/IITA), with tolerance or resistance to *Striga* and maize streak virus (MSV), and/or tolerance to drought (S1 Table). The inbred lines were extracted from twelve broad-based and two narrow-based source populations developed from both local and exotic germplasm identified based on several years of extensive testing for adaptation to the Guinea and Sudan savanna agro-ecologies of WCA (Table 1). The first generation of inbred lines were obtained following six generations of self-pollination in four early (TZE-W Pop DT STR C0, TZE-Y Pop DT STR C0, TZE Comp5-Y C6 and WEC STR) and two extra-early (TZEE-W Pop DT STR C0 and TZEE-Y Pop DT STR C0) broad-based populations with varied levels of drought tolerance and *Striga* resistance. A few inbred lines extracted from the two extra-early source populations, were categorized as early maturing because the flowering dates were typical of lines of the early maturing group. A second generation of inbred lines was developed from two F_2_ populations derived from the bi-parental crosses, TZEI 1 x TZEI 2 and TZEI 11 x TZEI 8 which involved elite parental inbred lines from two of the four broad-based early maturing populations. Another set of inbred lines was extracted from two other bi-parental crosses, TZE-W Pop x 1368 STR and TZE-W Pop x LD. The second generation of inbred lines was in all cases obtained following 6–7 cycles of self-pollination and selection for drought tolerance and/or resistance to *Striga*.

**Table 1:** Source populations of 94 maize inbred lines used in the present study.

S/N	Source population	Number of extracted inbred line	Grain colour
1	TZE-W Pop x 1368 STR	4	White
2	TZE-W Pop x LD	1	White
3	TZE-W Pop STR 108	10	White
4	TZE-W Pop STR Co	4	White
5	TZE-W P_OP_ STR 104	10	White
6	TZEE-W P_OP_ STR 104	4	White
7	TZEE-W P_OP_ STR 108	1	White
8	WEC STR	2	White
9	(TZEI 1 x TZEI 2)	10	White
10	TZE-Y Pop STR Co	12	Yellow
11	TZE-Y Pop STR 106	5	Yellow
12	TZEE-Y Pop STR 106	9	Yellow
13	TZE Comp5-Y C_6_	3	Yellow
14	(TZEI 11 x TZEI 8)	19	Yellow
	**Total**	**94**	

### DNA extraction and genotyping

Fresh leaves samples were collected from three weeks old seedlings within each genotype and stored in a deep freezer at -80°C. Prior to genomic DNA extraction, each sample was dried in a Labconco Freezone 2.5L System lyophilizer (Marshall Scientific, USA) followed by grinding using Spex^TM^ Sample Prep 2010 Geno/Grinder (Thomas Scientific, USA). Total genomic DNA extraction was performed using the DArT protocol (www.diversityarrays.com/files/DArT_DNA_isolation.pdf). The quality and quantity of DNA in each sample was determined on 2% agarose gel followed by quantification using an ND-1000 Spectrophotometer (Nanodrop Technologies). For genotyping, DNA samples were sent to Diversity Arrays Platform [29]. Library construction, sequencing and SNP calling was performed at the Diversity Arrays Facility (Canberra, Australia).

### Statistics and population structure analysis

SNP markers with more than 20% of missing data, 20% of heterozygosity and the minor allele frequency lower than 0.05 were eliminated resulting in 15,047 SNP markers which were used for further analysis [30]. The polymorphic information content (PIC), major allele frequency, the number of alleles, heterozygosity and gene diversity were estimated with the aid of PowerMarker V3.2.5 [31].

The data from the 15,047 SNP markers were subjected to population structure analysis based on the admixture model-based clustering method using the software package STRUCTURE 2.3.4 [32]. The best k was identified by inputting the data into the STRUCTURE HARVESTER software utilizing the Evanno method [33,34]. The initial model was run by varying the number of clusters (k) from 1 to 20 with 10 to 20 alterations for each K. Finally, the number of clusters was set at 10 with 10 alterations for each K. The results were obtained by running the data against 10,000 Markov Chain Monte Carlo and 10,000 burn-in as previously described [30]. Each genotype was assigned to a cluster at a 90% threshold, while genotypes with less than this value were assigned to an additional cluster designated as mixed cluster.

### Cluster analysis

Following the determination of the number of clusters using STRUCTURE, the 15,047 SNP marker data were analyzed using DARwin software [35]. The neighbor-joining method (NJ) under 30,000 bootstraps was used. Genetic distance matrix was generated using the Jaccard similarity test in the DARwin software [36], using the formula dij = (b + c)/ (a+ (b +c)) where dij is the dissimilarity between units i and j, a is the number of variables where Xi is present and Xj is present, b is the number of variables where Xi is present and Xj is absent, c is the number of variables where Xi is absent and Xj is present. To generate the final phylogenetic tree, the results obtained from DARwin were loaded into FigTree version 1.4.3 software [37].

## Results

### Panel summary statistics

The results of the summary statistics of the SNP markers are presented in Table 2. Heterozygosity averaged 0.07 and varied from 0.00 to 0.20. Gene diversity ranged from 0.01 to 0.50 with an average of 0.22. The major allele frequencies of the 15,047 primers averaged 0.84 with a range from 0.50 to 0.99. The PIC ranged from 0.01 to 0.38 with an average of 0.19 (S2 Table). The highest and lowest minor allele frequency recorded were 0.50 and 0.01, respectively.

**Table 2:** Diversity indices statistics of the 94 maize inbred lines based on 15,047 SNP markers.

	MaF	GD	He	PIC	MAF
Minimum	0.50	0.01	0.00	0.01	0.01
Maximum	0.99	0.50	0.20	0.38	0.50
Mean	0.84	0.22	0.07	0.19	0.16

MaF = Major allele frequency, GD = gene diversity; He = Heterozygosity, PIC = polymorphic information content, MAF = Minor allele frequency

### Genetic distance and population structure using SNP markers

**Fig 1:**
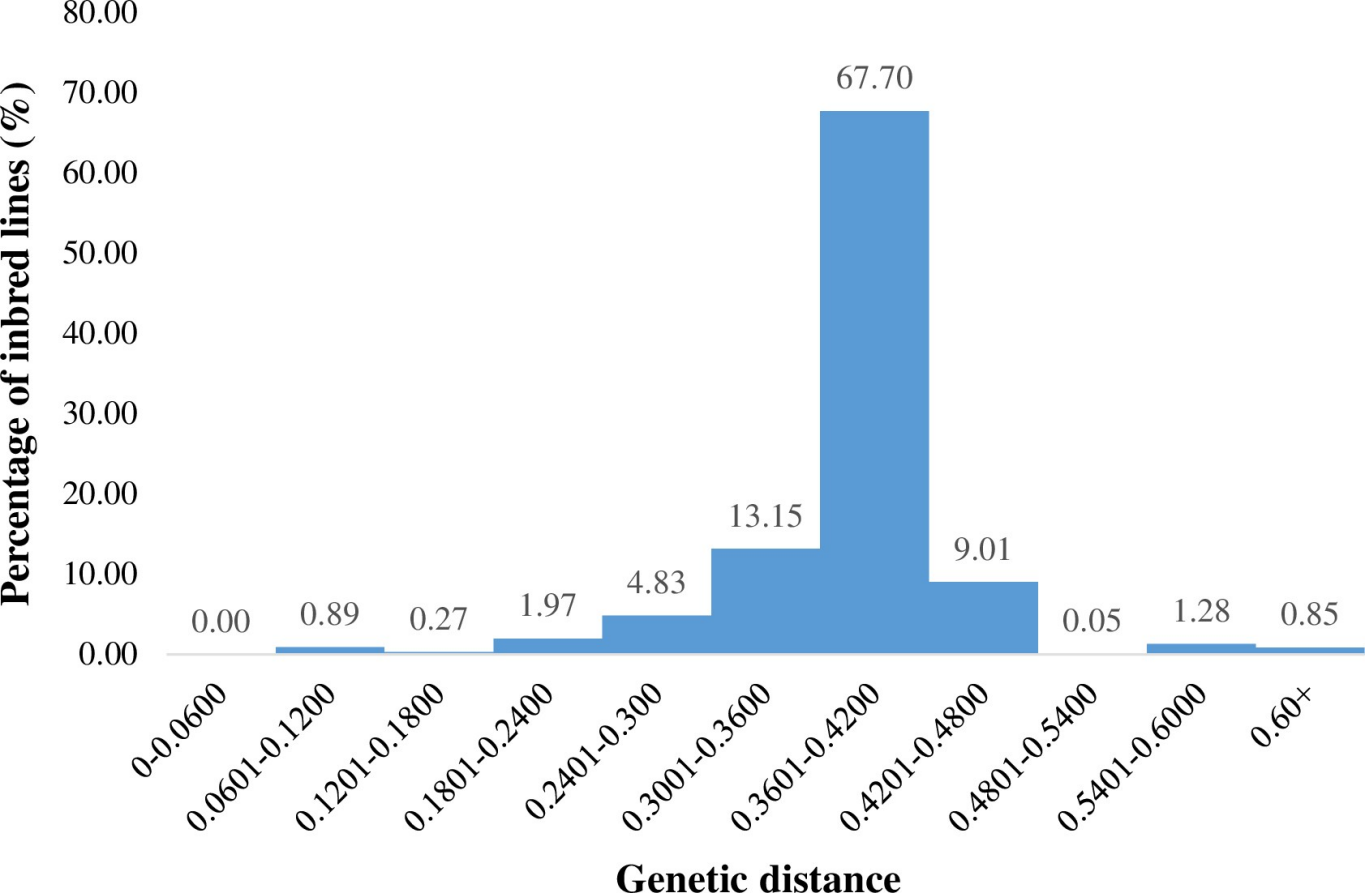
Frequency distribution of pairwise genetic distances calculated based on Euclidean method for 94 tropical maize inbred lines genotyped with 15,047 SNPs.

The genetic distance between pairwise comparisons of the inbred lines varied from 0.06 to 0.65, and the overall average distance was 0.38. The majority (67.70%) of the genetic distances fell between 0.32 and 0.42 (Fig 1). The lowest genetic distance (0.06) was observed between inbred TZdEI 24 and TZdEI 68 (S3 Table). Both inbred lines have the same descent and selection history (S1 Table). The highest genetic distance (0.65) was observed between inbred TZEI 135 and TZEI 26. These inbred lines were derived from different source populations; TZEI 26 was extracted from WEC STR, which has white endosperm colour, while TZEI 135 was derived from TZE-Y Pop STR C_0_, which has yellow endosperm colour (S1 Table)_._

**Fig 2:**
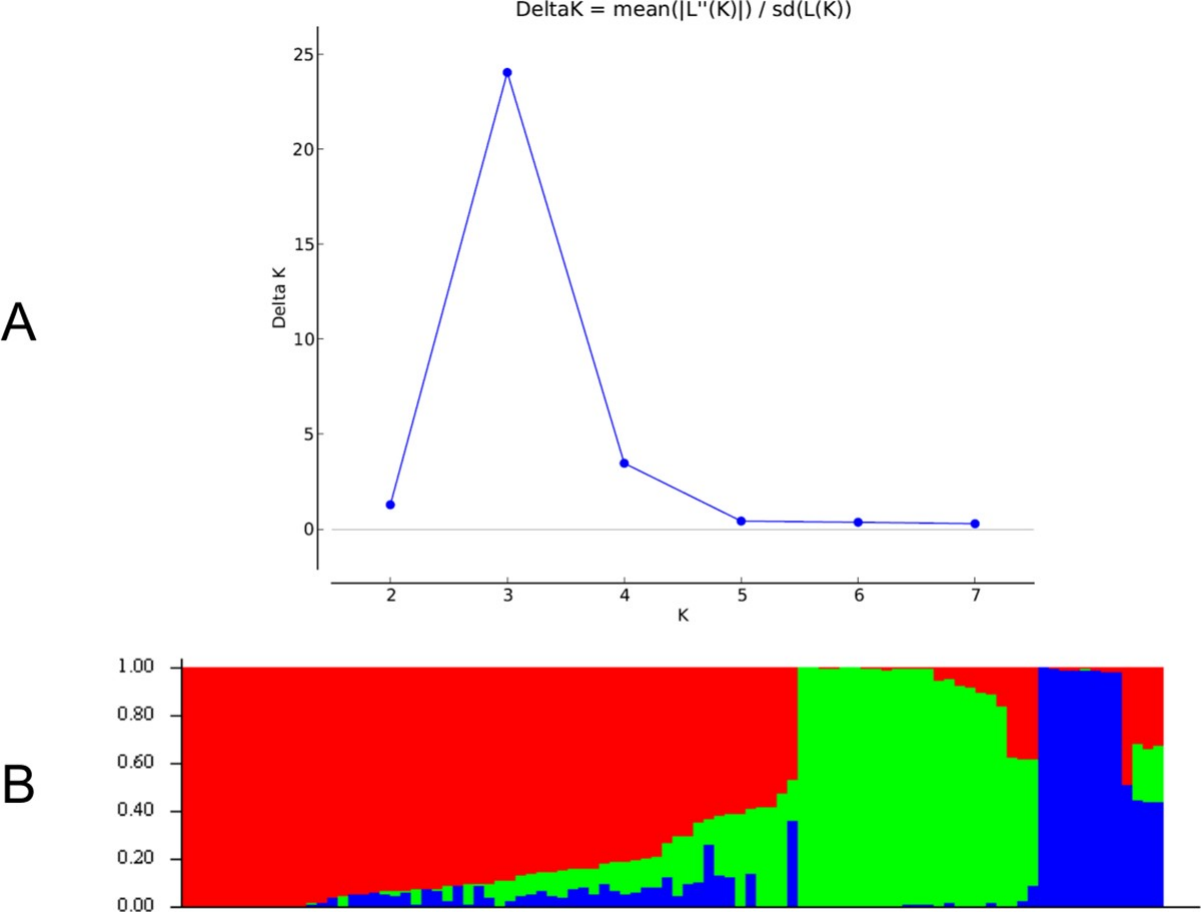
The three sub-populations of the 94 tropical maize inbred lines using SNP markers. A. Best delta K estimation by Evanno method. B. Estimated population structure of 94 tropical maize inbred lines as revealed by 15,047 SNP markers for K = 3. Blue, green and red colour represents sub-population 1, 2, and 3, respectively.

Based on 15,047 SNPs, population structure analysis revealed three distinct sub-populations in the 94 inbred lines (Fig 2A and 2B). The sub-population 1 consisted of 8.5% (8 lines) of the inbred lines, 20.2% (19 lines) were grouped into sub-population 2 and 36.1% (34 lines) in sub-population 3. A 35.1% (33 lines) of the inbred lines had a probability of association less than 90%, and were grouped into mixed populations (S1 Table). The three sub-populations were separated mainly on the basis of the endosperm color, sub-populations 1 and 2 comprising only yellow endosperm maize inbred lines while majority of inbred lines (94%) in sub-population 3 consisted of white endosperm maize (S1 Table). Results showed that sub-population 1 contained only inbred lines derived from the source population TZEE-Y Pop STR 106 while the sub-population 2 comprised about 97% of inbred lines extracted from (TZEI 11 x TZEI 8). Sub-population 3 had the greatest diversity comprising six diverse source populations, namely TZE-W Pop STR 108, TZE-W Pop STR 104, (TZEI 1 x TZEI 2), TZE-W Pop x 1368 STR, WEC STR and TZE-W Pop STR Co. The expected heterozygosity among inbred lines within the three sub-populations ranged between 0.06 for sub-population 1 and 0.34 for sub-population 3 with an average of 0.19. Sub-populations 1, 2 and 3 had F_ST_ values of 0.83, 0.49 and 0.01, respectively. The highest allele frequency divergence of 0.15 was recorded between sub-populations 2 and 1, followed by sub-population 3 and 1 with 0.11, while the least allele frequency divergence of 0.07 was recorded between sub-populations 2 and 3.

### Cluster analysis

**Fig 3:**
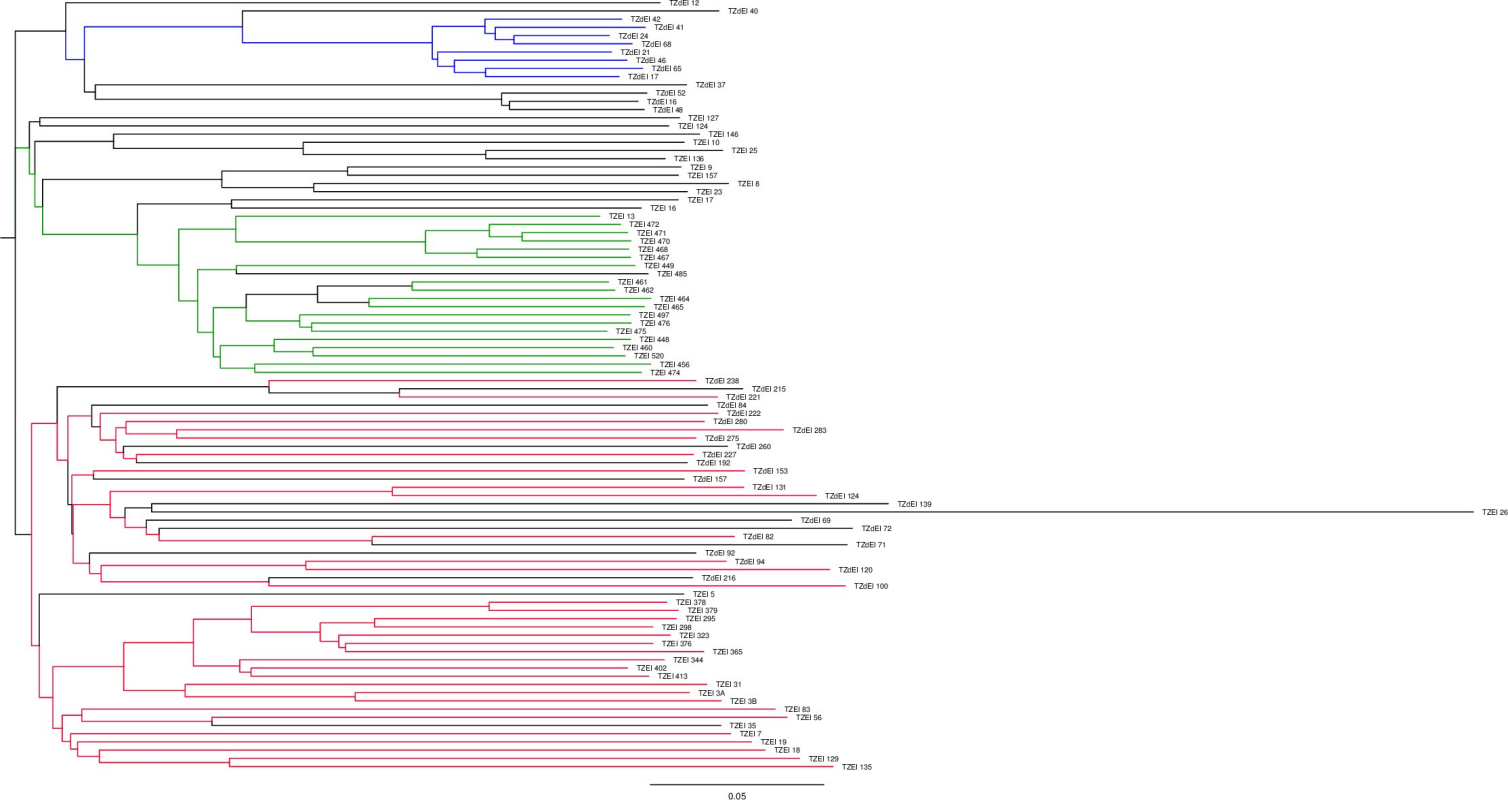
Clustering of 94 tropical maize inbred lines based on 15,047 SNP markers. Clusters resulting from structure analysis are shown in blue, green and red colours. Mixed individuals that did not belong to any particular group at a 90% threshold are represented by the black colour.

The results of the population structure analysis were confirmed by the phylogenetic tree which showed that the 94 inbred lines genotyped formed three clusters and each cluster was further partitioned into sub-clusters (Fig 3). In each cluster, inbred lines were coloured based on the results of the population structure analysis. Each colour represents specific sub-population such as lines coloured with blue, green, red and black corresponding to sub-population (SP) 1, 2, 3 and 4, respectively. The sub-population 4 represents the mixture of individuals at a 90% threshold. The inbred lines were clustered based on their pedigree, selection history and endosperm colour (S1 Table). Phylogenetic cluster 1 had a total of 14 inbred lines and represented a very clear sub-cluster of 8 lines (blue colour). This sub-cluster corresponded to sub-population 1 in population structure analysis (Fig 2B), with the remaining lines considered as mixed genotypes. All 14 lines were derived from the TZE-Y P_OP_ STR 106 and were characterized by yellow endosperm maize lines. The second cluster generated by the phylogenetic analysis included 32 inbred lines classified into several sub-clusters. However, it is clear that the grouping of 20 maize inbred lines based on the phylogenetic analysis was similar to that based on the population structure analysis (SP2; green color) (Figs 2B and 3). Ninety-five percent of the lines were extracted from the bi-parental cross TZEI 11 x TZEI 8 while one of the inbred lines was sourced from TZE Comp5-Y C_6_. The remaining 17 lines were derived from two source populations TZE-Y Pop STR Co and TZE Comp5-Y C_6_ and were identified as mixed genotypes by structure analysis. The third and last cluster was the largest and most diverse among the three main clusters with 48 maize inbred lines. This cluster included inbred lines from seven source populations, namely TZE-W P_OP_ STR 108, TZE-W P_OP_ STR 104, TZE-W Pop x 1368 STR, TZE-W Pop x LD, (TZEI 1 x TZEI 2), WEC STR and TZE-Y Pop STR Co (S2). All the inbred lines in this phylogenetic cluster belong to sub-population 3 (red colour) except 12 lines which were classified by the population analysis into the admixture group (Fig 3).

## Discussion

Genetic diversity studies are very essential in the selection of individual genotypes among closely related groups for initiation of new breeding activities. It is particularly important that the genetic similarity or dissimilarity among inbred lines are established to facilitate the development of productive hybrids which are often a product of crosses involving inbred lines of opposing heterotic groups. Heterotic populations are also developed from inbred lines sharing common ancestry. New set of inbred lines are derived from the heterotic populations and the inbred lines are expected to combine well with inbred lines developed from other opposing heterotic populations. Genotyping has proved to be one of the reliable approaches of establishing such phylogenetic relationships among a set of inbred lines [8]. In the present study, the PIC average was of 0.19, a value found lower than ones described by some researchers in wheat (0.44; [38]), maize (0.25–0.39, [30,39–43]) and sugar beet (0.28; [44]), but similar to the results of Cruz et al. (0.21; [45]) in an oilseed crop Lesquerella, Bisen et al. [46] in soybean, Ramakrishnan et al. [47] in finger millet, Singh et al. [48] in rice and Oyekunle et al. [17], Dao et al. [40] and Senior et al. [49] in maize. Approximately 49% of the SNPs presented a MAF<0.1 and 4.5% of the SNPs showed almost equal allele frequencies (MAF ~0.5) for the two alternative alleles. Previously, it was shown in maize by using a set of 1,057 SNPs that IITA maize materials presented an average value of 0.218 and 0.202 for PIC and MAF values, respectively. Similar values were found for CIMMYT maize inbred lines [40]. The variation between our results and the findings of earlier researchers could be attributed to the differences in the composition of experimental material, population size and number of markers involved in the studies [50,51].

Genetic distance provides a measure of the degree of relatedness between individuals in a population [52]. The results obtained revealed a wide genetic variability among the inbred lines with lower number of pairwise individuals with low genetic distances, suggesting that most of the inbred line used are unique and each of them has the potential to contribute new alleles to the breeding programme.

The SNP markers clustered the inbred lines based on their ancestry, selection history and endosperm colour (Figs 2 and 3; S1 Table). However, the clustering of some inbred lines was not based on shared ancestry, indicating that inbred lines extracted from the same source population do not necessarily have the same selection history [53]. The lack of association between clustering patterns and phenotypes, environmental adaptation, maturity and the heterotic groups has also been previously observed in CIMMYT maize germplasm [51,54]. Warburton et al. [54] suggested that markers may be better indicators of relatedness of the inbred lines in cases where inbreds derived from the same source population are more different than those extracted from different source populations.

Population structure analysis is a process of inferring individual ancestry of inbred lines from genotypic information [55]. The studied SNPs revealed the presence of three sub-populations (K = 3) within the 94 inbred lines. Inbred lines with similar pedigree tended to cluster into the same group. The SNP markers clearly assigned inbred lines into heterotic groups based on the source populations, with individuals of the same endosperm colour and similar genetic background placed in the same sub-population. The grouping of inbred lines using SNP markers on the basis of similarity of pedigree and phenotype indicated that the SNP markers were very effective in assigning the inbred lines into homogenous groups. Our findings might have been influenced by the relatively larger number of SNPs used in the present study which would facilitate better interpretation of the results and inference about population structure [51].

The lower levels of heterozygosity observed among inbred lines within the three sub-populations suggested that the SNPs were effective in forming homogeneous sub-populations. The very large F_ST_ values obtained for sub-populations and the moderate allele frequency divergence observed between sub-populations indicates that these inbred lines are fixed and can be classified into genetically distinct groups (heterotic groups). These characteristics make them a valuable resource for genetic studies in maize and association mapping where uniformity of inbred lines and genetic divergence are required. For future development of productive hybrids, crosses should be made between parent lines from different sub-populations, particularly between inbred lines in sub-populations 1 and 2.

## Conclusion

High genetic distances obtained among paired inbred lines revealed the uniqueness of the studied lines and existence of substantial genetic variability that could be exploited for the development of productive hybrids. The inbred lines were assigned into heterotic groups based on similarity of ancestry, selection history and endosperm colour. This study showed that SNP markers were more reliable in categorizing maize inbred lines into groups on the basis of shared phylogeny. The low heterozygosity observed among inbred lines within sub-populations and the moderate divergence among sub-populations suggested that the inbred lines could be used in the development of productive hybrids breeding or heterotic populations for the West African Sub-region.

## Supporting information

S1 Table Pedigree of 94 inbred lines and their summary of clustering and population structure using 15,047 SNP markers. (XLSX)

S2 Table Summary of 15,047 SNP markers used in present study. (XLSX)

S3 Table Genetic distance matrix of 94 inbreds used in present study. (XLSX)
